# DUSP12 regulates the tumorigenesis and prognosis of hepatocellular carcinoma

**DOI:** 10.7717/peerj.11929

**Published:** 2021-08-03

**Authors:** Gaoda Ju, Tianhao Zhou, Rui Zhang, Xiaozao Pan, Bing Xue, Sen Miao

**Affiliations:** 1 Department of Medical Oncology, Beijing Cancer Hospital, Peking University, Beijing, China; 2 Shanghai First People’s Hospital, Shanghai Jiao Tong University School of Medicine, Shanghai, China; 3 Department of Pathology, Affiliated Hospital of Jining Medical University, Jining, China

**Keywords:** Hepatocellular carcinoma, DUSP12, Mutation, Tumorigenesis, Prognosis

## Abstract

**Background:**

Dual specificity protein phosphatase (*DUSP)12* is an atypical member of the protein tyrosine phosphatase family, which are overexpressed in multiple types of malignant tumors. This protein family protect cells from apoptosis and promotes the proliferation and motility of cells. However, the pathological role of *DUSP12* in hepatocellular carcinoma (HCC) is incompletely understood.

**Methods:**

We analyzed mRNA expression of *DUSP12* between HCC and normal liver tissues using multiple online databases, and explored the status of *DUSP12* mutants using the cBioPortal database. The correlation between *DUSP12* expression and tumor-infiltrating immune cells was demonstrated using the Tumor Immune Estimation Resource database and the Tumor and Immune System Interaction Database. Loss of function assay was utilized to evaluate the role of *DUSP12* in HCC progression.

**Results:**

*DUSP12* had higher expression along with mRNA amplification in HCC tissues compared with those in normal liver tissues, which suggested that higher *DUSP12* expression predicted shorter overall survival. Analyses of functional enrichment of differentially expressed genes suggested that *DUSP12* regulated HCC tumorigenesis, and that knockdown of *DUSP12* expression by short hairpin (sh)RNA decreased the proliferation and migration of HCC cells. Besides, *DUSP12* expression was positively associated with the infiltration of cluster of differentiation (CD)4+ T cells (especially CD4+ regulatory T cells), macrophages, neutrophils and dendritic cells. *DUSP12* expression was positively associated with immune-checkpoint moieties, and was downregulated in a C3 immune-subgroup of HCC (which had the longest survival).

**Conclusion:**

These data suggest that *DUSP12* may have a critical role in the tumorigenesis, infiltration of immune cells, and prognosis of HCC.

## Introduction

Hepatocellular carcinoma (HCC) is the most common malignant tumor of the liver (Ferlay et al., 2015). HCC is the third most prevalent cause of cancer-specific death worldwide (Bray et al., 2018). Due to rapid progression, HCC is usually discovered and diagnosed at an advanced stage, which leads to the loss of feasibility of treatments (Chen et al., 2016). Systemic chemotherapy for HCC is limited because HCC lacks sufficient targets for drugs, and HCC evolves resistance to classic anti-tumor agents (Fitzmorris et al., 2015; Lohitesh, Chowdhury & Mukherjee, 2018). The median duration of survival of patients with advanced HCC is ∼1 year (Llovet et al., 2018; Zhu et al., 2015).

Dual specificity protein phosphatase (*DUSP)12* is an atypical member of the protein tyrosine phosphatase (PTP) family. *DUSP12* regulates the proliferation, apoptosis, and migration of cells by dephosphorylating tyrosine and serine/threonine residues (Guan, Broyles & Dixon, 1991; Patterson et al., 2009). It has been reported that *DUSP12* is overexpressed in intracranial ependymoma, retinoblastomas, and neuroblastomas (Gratias et al., 2005; Hirai et al., 1999; Mendrzyk et al., 2006). Some research teams have found that DUSP12 overexpression protects HeLa cells from apoptosis and promotes the proliferation and motility of HEK293 cells (Cain, Braun & Beeser, 2011; Sharda et al., 2009). Several studies have reported that *DUSP12* overexpression in macrophages could reduce expression of proinflammatory cytokines such as tumor necrosis factor- *α*, interleukin (IL)-1 and IL-6, and increase IL-10 expression. *DUSP12*-expressed hepatocytes are less inflamed and cause less hepatic steatosis than *DUSP12*-deleted hepatocytes (Cho et al., 2017; Huang et al., 2019). However, the correlation between *DUSP12* expression and HCC tumorigenesis and *DUSP12* function in cells is not known.

We explored the expression, mutation, and pathological role of *DUSP12* in HCC by integrated analyses of various data sources using online tools. The latter were applied to analyze the correlation of target genes with different cancer types, but especially HCC. In this way, we hoped to help researchers investigate the molecular targets of tumorigenesis.

## Materials & Methods

### Public databases

#### Ualcan

Ualcan (http://ualcan.path.uab.edu/) is an interactive Internet resource for analyzing cancer OMICS data. Ualcan was used to analyze transcription expression, prognosis, and the methylation of genes in The Cancer Genome Atlas (TCGA) datasets (Chandrashekar et al., 2017). The TCGA-Liver Hepatocellular Carcinoma (LIHC) dataset was employed in our research.

#### Gene expression profiling interactive analysis (GEPIA)

The GEPIA database (http://gepia.cancer-pku.cn/) was used to plot overall survival (OS) and disease-free survival (DFS) curves. Group cutoff was based on the median of gene expression and prognostic status of patients in the TCGA-LIHC dataset (Tang et al., 2017).

#### Kaplan–Meier Plotter (liver cancer)

Kaplan–Meier Plotter (http://kmplot.com/analysis/index.php/) was employed for OS (including 364 patients), DFS (including 316 patients), progression-free survival (PFS) (including 370 patients) and disease-specific survival (DSS) (including 262 patients) analysis using data from RNA-sequencing of a liver-cancer dataset (Menyhárt, Nagy & Gyorffy, 2018). We separated high and low expression based on the best cutoff expression value of *DUSP12* that all possible cutoff values between lower and upper quartiles were computed, and the best performing threshold was used as a cutoff.

#### Human protein atlas (HPA)

The HPA database (http://www.proteinatlas.org/) was used to validate gene expression in liver-cancer tissues and normal liver tissues at the protein level (Uhlén et al., 2015; Uhlen et al., 2017).

#### Cancer cell line encyclopedia

The Cancer Cell Line Encyclopedia database (http://www.DepMap Broadinstitute.org/ccle/) was employed to analyze gene expression in HCC cells (Ghandi et al., 2019). RNA-expression data of liver-cancer cell lines were downloaded from this website for our research.

#### HCCDB

The Hepatocellular Cancer Database (HCCDB) (http://lifeome.net/database/hccdb/home.html) is an integrative molecular database of HCC with 15 datasets. Co-expressed genes were computed and displayed in HCC according the guidelines on the HCCDB website (Lian et al., 2018). The normalized expression data of ICGC-LIRI-JP and GSE14520 were downloaded from HCCDB.

#### cBioPortal

The cBioPortal database (http://cbioportal.org/) is an online tool for analyzing the mutation characteristics of genes in a Liver Hepatocellular Carcinoma (TCGA, Firehose Legacy) dataset (Cerami et al., 2012; Gao et al., 2013). A total of 392 differentially expressed genes (DEGs) were identified from a *DUSP12*-altered group and *DUSP12*-nonaltered group of patients.

#### Tumor immune estimation resource (TIMER)

The correlation of copy number variation (CNV) of genes with the abundance of tumor-infiltrating immune cells (TIICs) was displayed by an online tool in the TIMER database (Li et al., 2016; Li et al., 2017). In this way, we analyzed the correlation of gene expression with TICC abundance and expression of immune-checkpoint moieties in the TCGA-LIHC dataset (https://cistrome.shinyapps.io/timer/), the list of correlations was filtered for interactions with *P* <0.05 and correlation coefficient >0.2.

#### Tumor and immune system interaction database (TISIDB)

TISIDB (http://cis.hku.hk/TISIDB/) was employed to analyze gene expression in patients with different immune subtypes of HCC (Ru et al., 2019).

#### CIBERSORT

A total of 369 tumor samples extracted from the Genomic Data Commons (GDC)-TCGA-LIHC dataset were downloaded from UCSC.XENA (http://xena.ucsc.edu/). The CIBERSORT method was used within the R package (http://www.r-project.org/) (Goldman et al., 2020; Newman et al., 2019). After removing samples with *P* ≥ 0.05 in the result of CIBERSOT analysis, the remaining 261 samples were divided into high and low expression groups based on median expression of *DUSP12*. Bar graphs of the TIIC ratio between high and low expression groups were plotted with Prism 7 (GraphPad, San Diego, CA, USA).

#### Metascape

Metascape (http://metascape.org/) is a resource to aid the annotation and analyses of genes, which helps biologists make sense of one or multiple gene lists. Metascape was applied for analyses of protein–protein interaction (PPI) networks. Analyses of functional enrichment and enrichment of pathways were done using Gene Ontology (GO), Kyoto Encyclopedia of Genes and Genomes (KEGG) and DisGeNET databases (Zhou et al., 2019).

#### Depmap portal database

DepMap Portal (https://depmap.org/portal/) database is utilized to evaluate the probabilities of dependency of *DUSP12* in HCC cell lines with CERES score based on data from CRISPR (DepMap 21Q2 Public + Score, CERES) cohort (DepMap Broad, 2021).

### Cell lines and culture conditions

The human liver-cancer cell line Huh7 was acquired from American Type Culture Collection (Manassas, VA, USA) and cultivated in Dulbecco’s modified Eagle’s medium (DMEM; Gibco, Grand Island, NY, USA) with 10% fetal bovine serum (Gibco) and 1% penicillin–streptomycin (Gibco) at 37 °C in an atmosphere of 5% CO_2_.

### Western blotting

Cell proteins were extracted by denaturing buffer and then quantified by a bicinchoninic acid protein assay (Thermo Scientific, Waltham, MA, USA). Protein lysates from the HCC cell line were separated by sodium dodecyl sulfate–polyacrylamide gel electrophoresis, transferred to nitrocellulose membranes (Millipore, Bedford, MA, USA), blocked, and then detected by primary antibody *DUSP12* (1:2000 dilution; catalog number: ab237008; Abcam, Cambridge, UK) and horseradish peroxidase-conjugated secondary antibody (Sigma–Aldrich, Saint Louis, MO, USA). These actions were followed by exposure to enhanced chemiluminescence. The housekeep gene *β*-tubulin (1:500; ab6046; Abcam) was used as a loading control.

### Plasmids and lentivirus production

Annealing and connection of short hairpin (sh)RNA were undertaken followed by construction into the modified plasmid pLKO.1. Well-constructed vectors were transinfected into HEK293T cells by lentivirus packaging plasmids psPAX and pMD2.0G. The shRNA sequences and *DUSP12* primer sequence (forward and reverse, respectively) were: CCGGGTTGAGTGGCAACTGAAATTATCTCGAGATAATTTCAGTTGCCACTCAAGTTTTTG, and AATTCAAAAAGTTGAGTGGCAACTGAAATTATCTCGAGATAATTTCAGTTGCCACTCAAG for sh*DUSP12*-1; CCGGGTGGATACCTCTAGTGCAATTCTCGAGAATTGCACTAGAGGTATCCACTTTTTG, and AATTCAAAAAGTGGATACCTCTAGTGCAATTCTCGAGAATTGCACTAGAGGTATCCAC for sh*DUSP12*-2.

### Cell-growth assay

Lentivirus-infected stable cells were seeded into 96-well plates and cultured in DMEM containing 10% fetal bovine serum (2000 cells per well, five parallel wells). Then, cells were collected at different timepoints. The cell number in each well was counted by Cell Counting Kit 8 (CCK8). Absorbance at 450 nm was measured to determine of the number of viable cells.

### Transwell™ assay

Lentivirus-infected stable cells were seeded in the upper chamber of a Transwell chamber (24-well (8-µm pore; Corning, Corning, NY, USA) in 200 µL of serum-free DMEM (1 ×10^5^ cells per well, five parallel wells). Then, 800 µL of DMEM containing 10% fetal bovine serum was added to the lower chamber and incubation allowed for 36 h at 37 °C. After removing the cells at the upper surface of the membrane, cells were passed through a filter and fixed with 4% paraformaldehyde, stained with 0.1% Crystal Violet solution and photographed using an inverted fluorescence microscope.

## Results

### Pattern of *DUSP12* transcriptional expression using Ualcan and HCCDB databases

**Figure 1:**
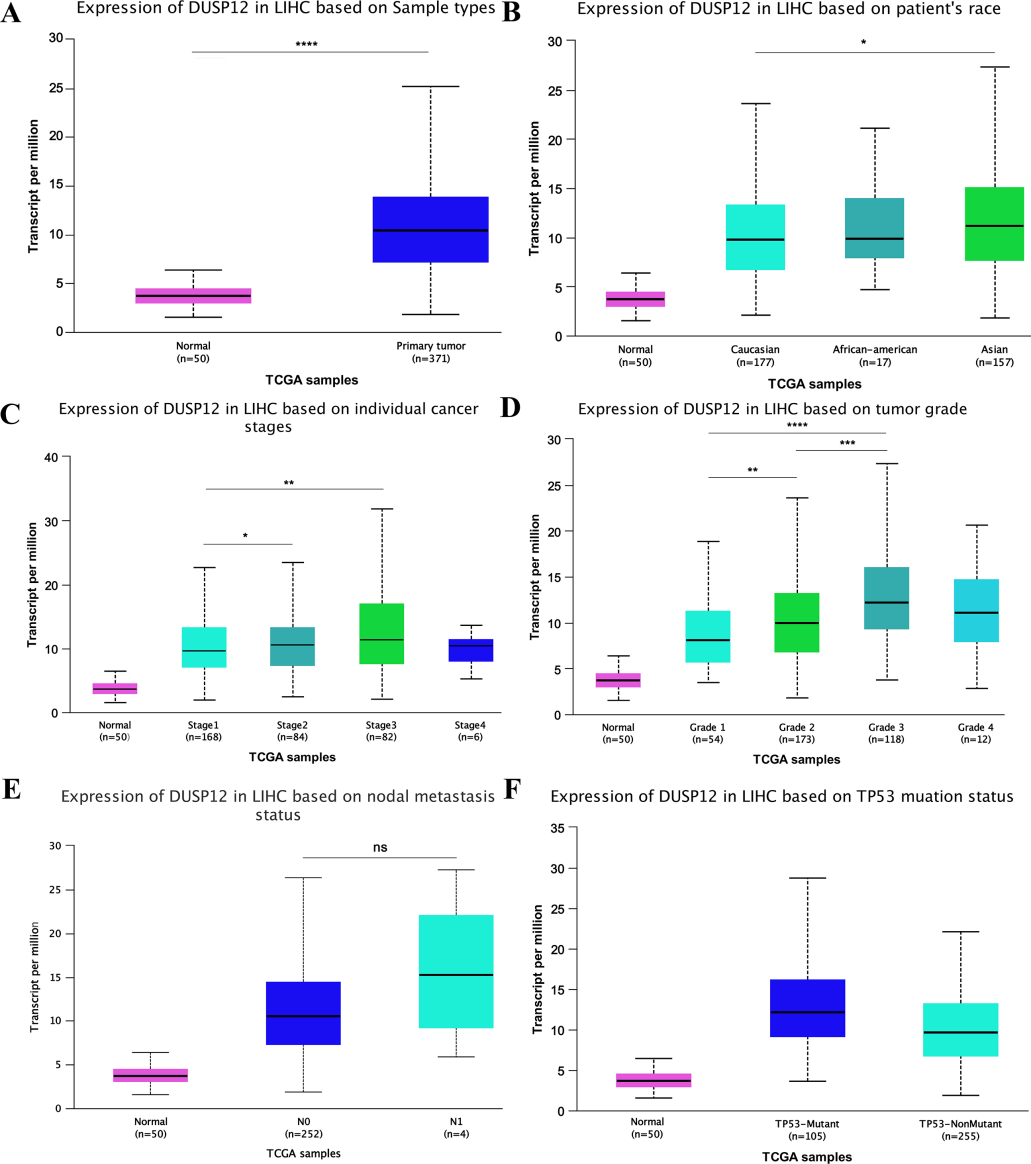
*DUSP12* expression in the Ualcan database (A) Normal *vs*. primary tumor. (B) Ethnicity. (C) Stage. (D) Grade. (E) Nodal metastasis. (F) TP53 mutation. ^∗^*P* < 0.05, ^∗∗^*P* < 0.01, ^∗∗∗^*P* < 0.001, ^∗∗∗∗^*P* < 0.0001.

**Figure 2:**
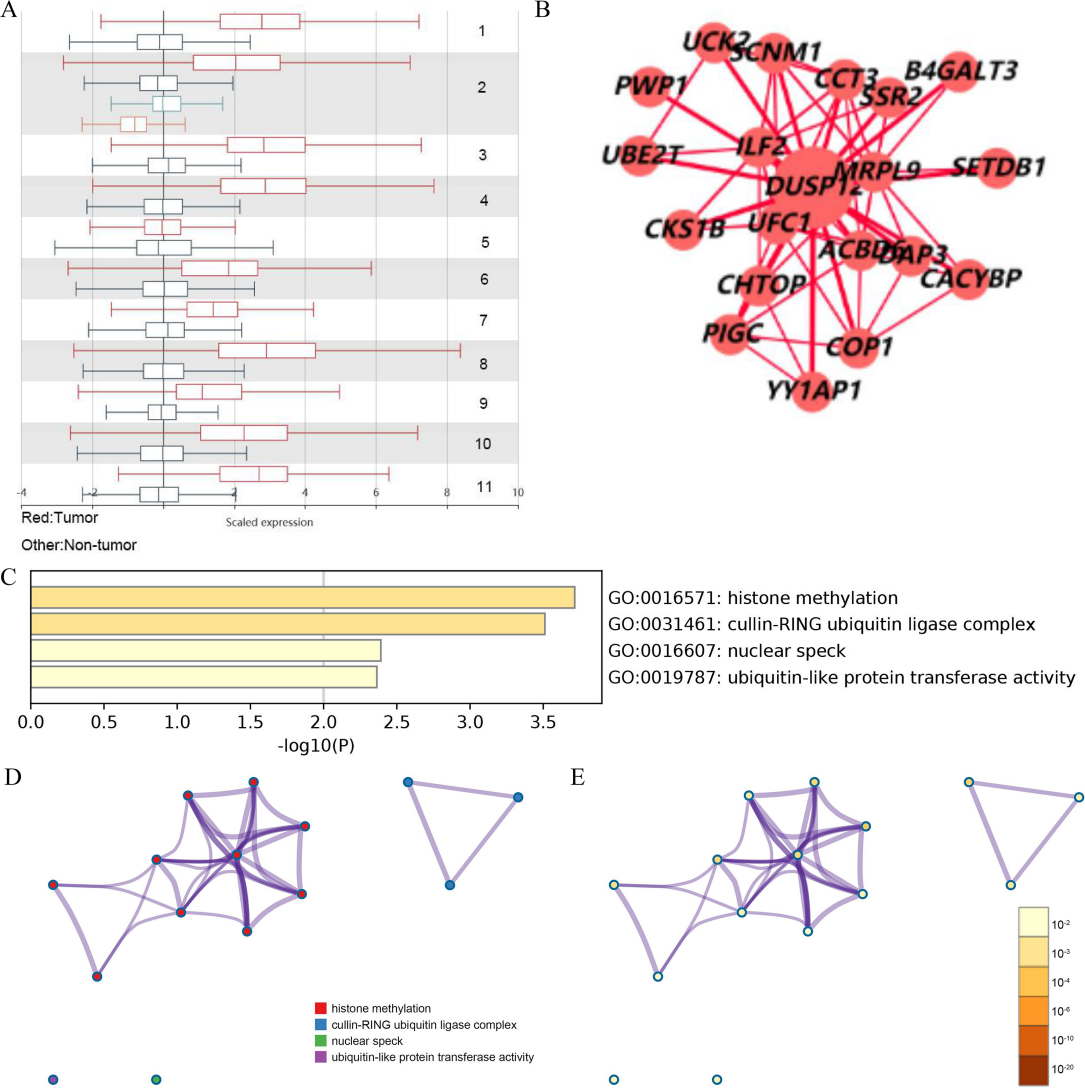
Analysis of gene expression in the HCCDB (A) *DUSP12* expression in 11 cohorts. (B) PPI network containing *DUSP12*. (C) Heatmap for selected genes in the GO database (*P* < 0.05). (D) Network colored by cluster. (E) Network colored by *P*-value. GO, gene ontology; PPI, protein-protein network.

Analyses of the TCGA-LIHC dataset in the Ualcan database and analyses of ICGC-LIRI-JP and GSE14520 cohorts revealed that transcriptional expression of *DUSP12* was higher in LIHC tissues compared with that in normal liver tissues (Fig. 1A, Fig. S1). Analyses of clinical subgroups demonstrated that *DUSP12* expression was higher in an Asian, tumor–node–metastasis (TNM) stage-III, grade-3, P53-mutant group than that in a Caucasian, TNM stage-I/II, grade-1/2, P53-nonmutant group. The non-significant change in *DUSP12* expression between the stage-IV/grade-4/N1 group compared with that in other groups may have been caused by the number of samples in the stage-IV/grade-4/N1 group being significantly less compared with that in other groups. The top positively and negatively correlated genes in TCGA dataset for DUSP12 were also downloaded from UALCAN database (Figs. S1 and S2). We suspected that *DUSP12* could be a pathological and prognostic marker of LIHC (Figs. 1B–1F). Integrative analyses of HCCDB revealed that mRNA expression of *DUSP12* was higher in liver-cancer tissue compared with that in normal liver tissue in 11 cohorts (Fig. 2A). Analyses of functional enrichment of genes co-expressed with *DUSP12* in liver-cancer tissues using Metascape showed that these genes were engaged mainly in “histone methylation”, “cullin RING ubiquitin ligase complexes”, “nuclear specks” and “ubiquitin-like protein transferase activity” (Figs. 2B–2E).

### Validation of *DUSP12* expression in tissues at the protein level and liver-cancer lines

**Figure 3:**
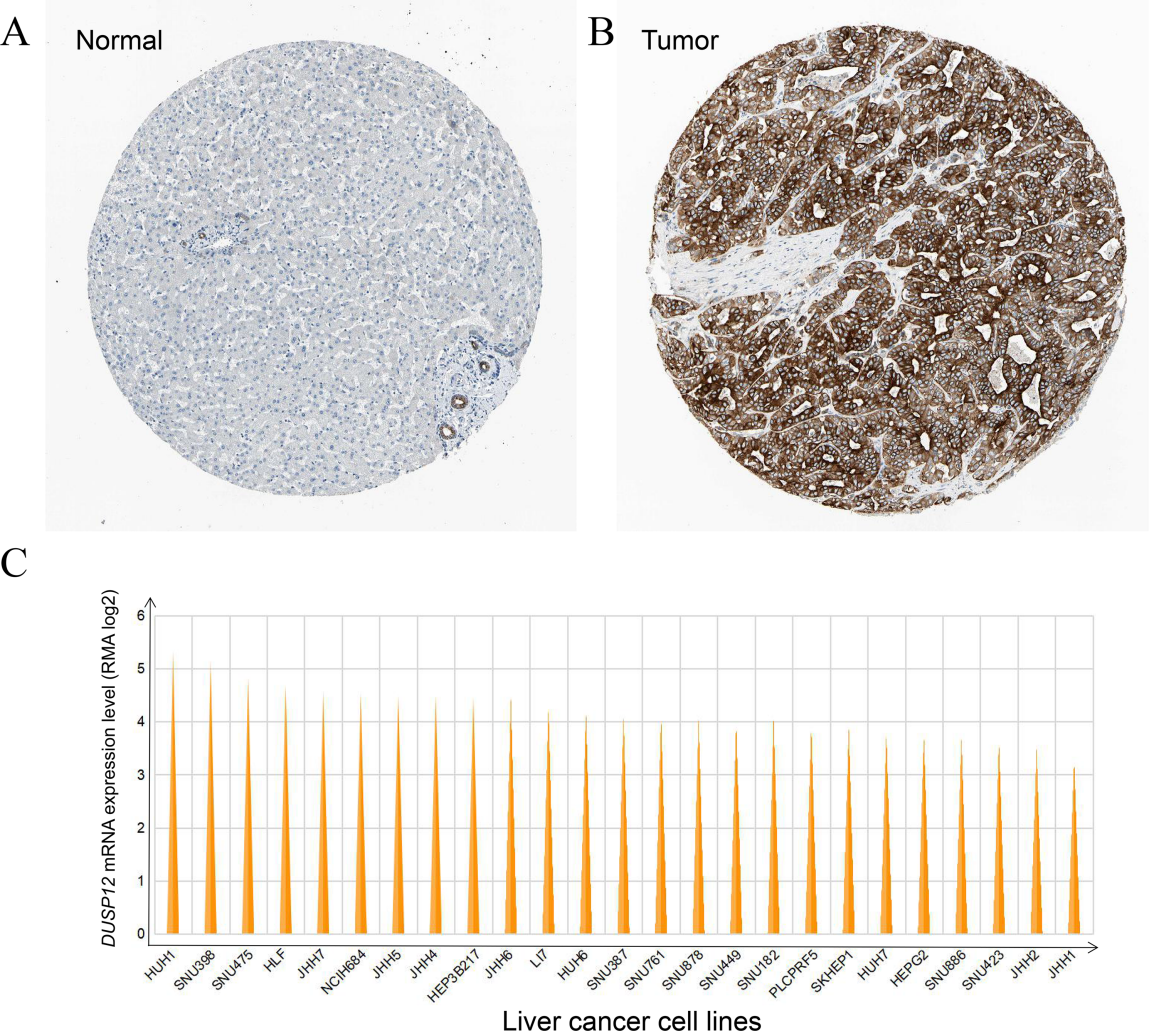
*DUSP12* expression in liver-cancer tissues, normal liver tissues, and liver-cancer cell lines. (A) Normal liver tissues. (B) Liver-cancer tissues. (C) Liver-cancer cell lines.

*DUSP12* expression was higher in liver-cancer tissues than that in normal liver tissues at the protein level, and this observation was validated by results from the HPA database (Antibody: HPA008840; cancer-patient ID: 2399; normal-patient ID: 3222) (Figs. 3A, 3B) and HUH1 had the highest transcriptional expression of *DUSP12* in liver-cancer cell lines (Fig. 3C).

### Survival analyses of liver-cancer patients with different *DUSP12* expression

**Figure 4:**
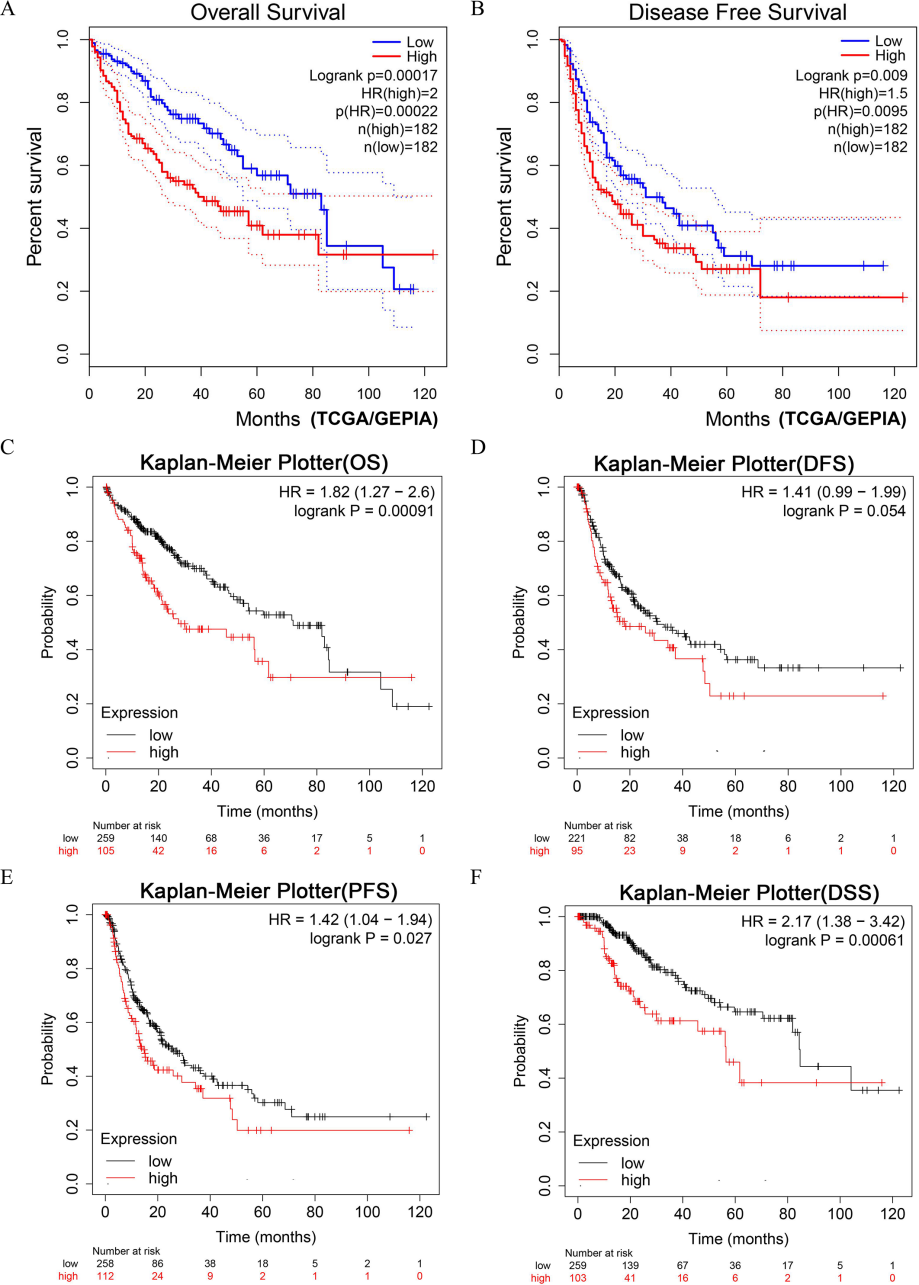
Survival analyses of HCC patients with high expression of *DUSP12* and low expression of *DUSP12*. (A) OS in GEPIA. (B) DFS in GEPIA. (C) OS in Kaplan–Meier Plotter. (D) DFS in Kaplan–Meier Plotter. (E) PFS in Kaplan–Meier Plotter. (F) DSS in Kaplan–Meier Plotter. OS, overall survival; DFS, disease free survival; PFS, progression free survival; DSS, disease specific survival; GEPIA, Gene Expression Profiling Interactive Analysis.

LIHC patients with higher expression of *DUSP12* had shorter OS and DFS in the TCGA-LIHC dataset (Figs. 4A, 4B). Next, we validated the result by survival analyses of liver-cancer patients in the Kaplan–Meier Plotter database. We showed that patients with higher expression of *DUSP12* had shorter OS, DFS, PFS and DSS than that of patients with lower expression of *DUSP12* (Figs. 4C–4F).

### Analyses of *DUSP12* expression using the cBioPortal database

**Figure 5:**
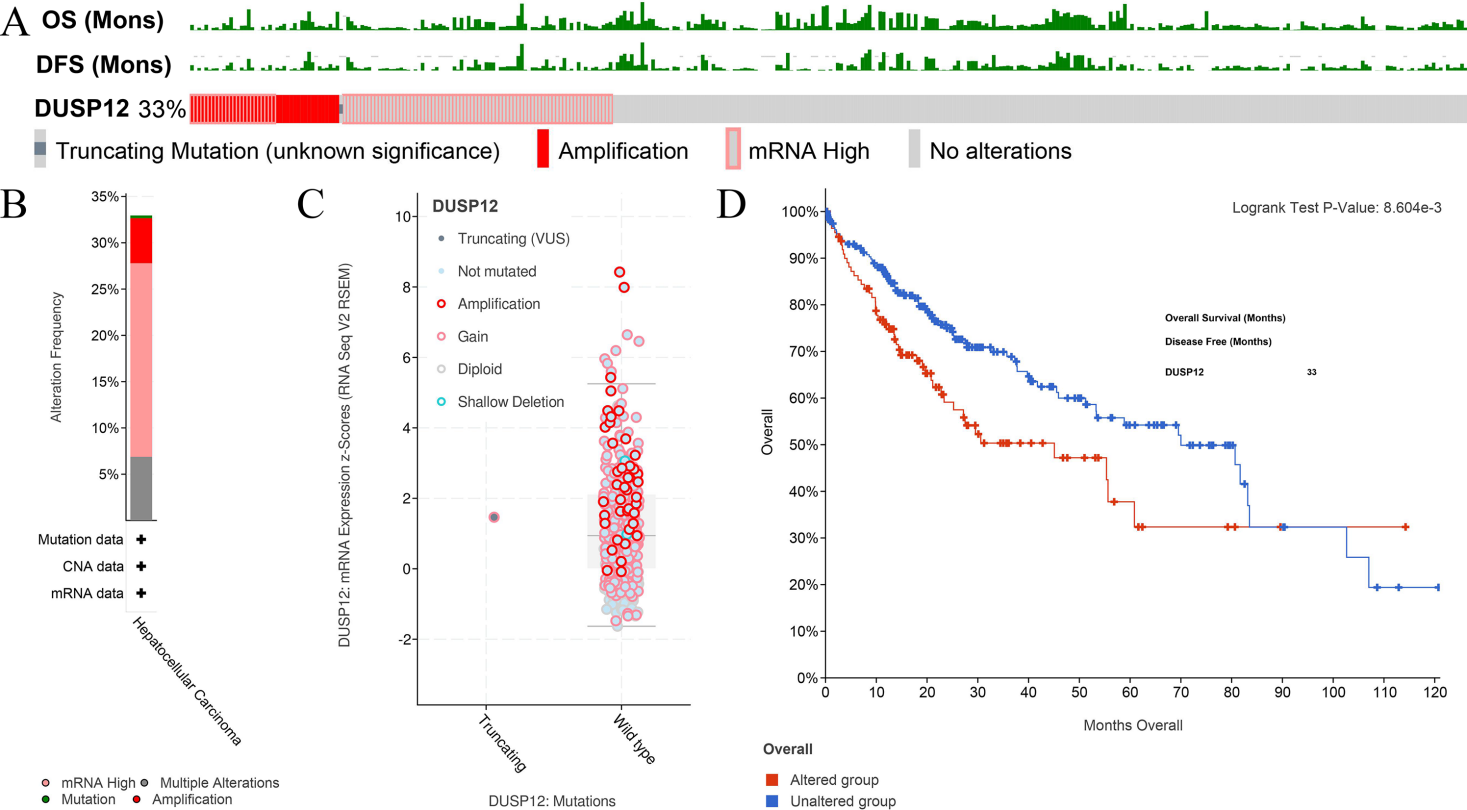
Analyses of mutant status of *DUSP12* in HCC. (A) Frequency of the *DUSP12* mutation. (B) Frequency of mutant types. (C) mRNA expression of *DUSP12* in HCC cases with various types of mutant status. (D) OS of *DUSP12*-altered and *DUSP12*-nonaltered groups. OS, overall survival.

**Figure 6:**
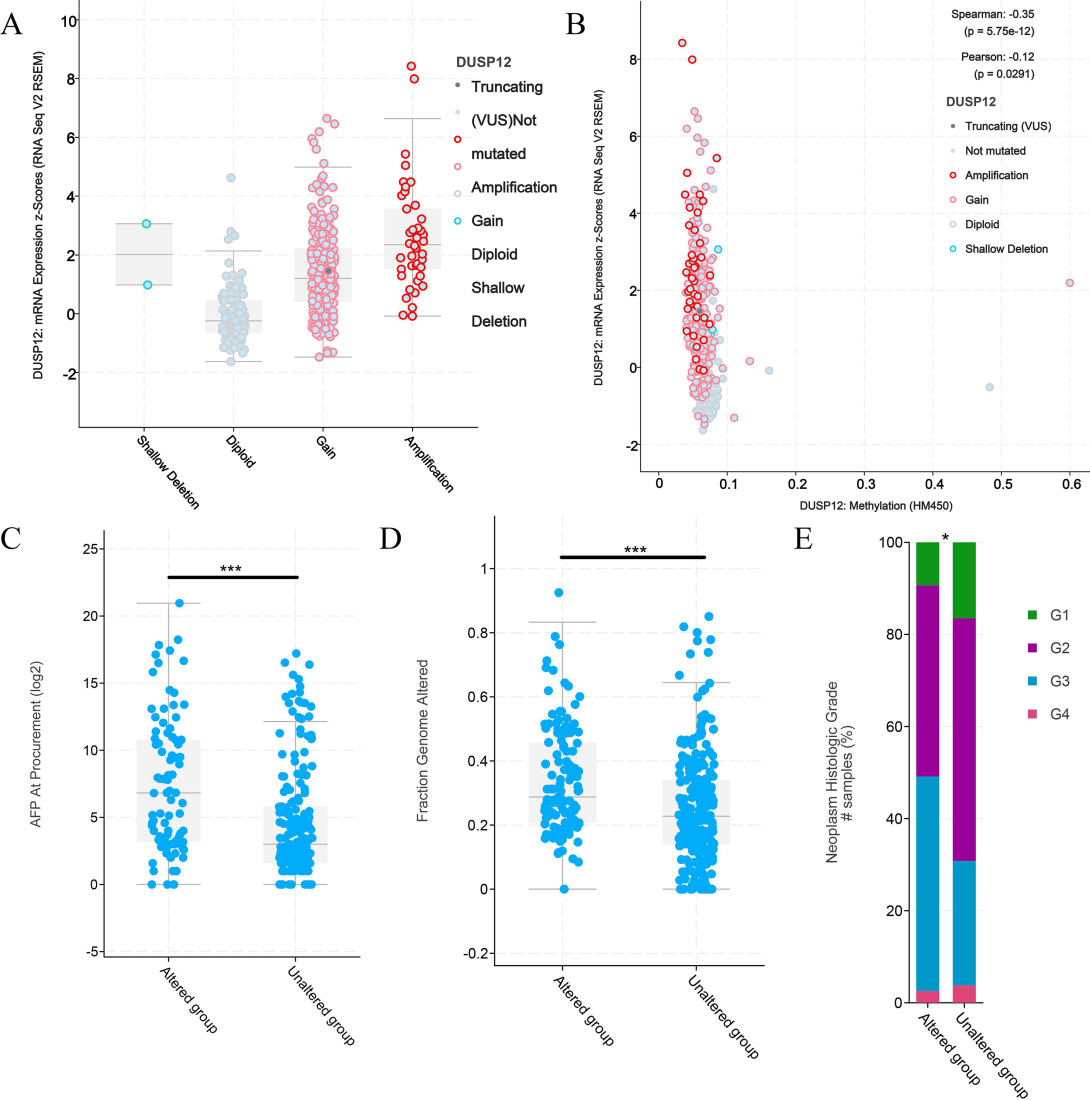
Clinical features of HCC patients with or without alteration of *DUSP12* expression. (A) mRNA expression of *DUSP12* in HCC patients with different copy numbers. (B) Relationship between mRNA expression of *DUSP12* and *DUSP12* methylation. (C) AFP level at procurement in *DUSP12*-altered and *DUSP12*-nonaltered groups. (D) Fraction of genome altered in *DUSP12*-altered and *DUSP12*-nonaltered groups. (E) Histology grade in *DUSP12*-altered and *DUSP12*-nonaltered groups. AFP, alpha-fetoprotein; ^∗^*P* < 0.05, ^∗∗∗^*P* < 0.001.

**Figure 7:**
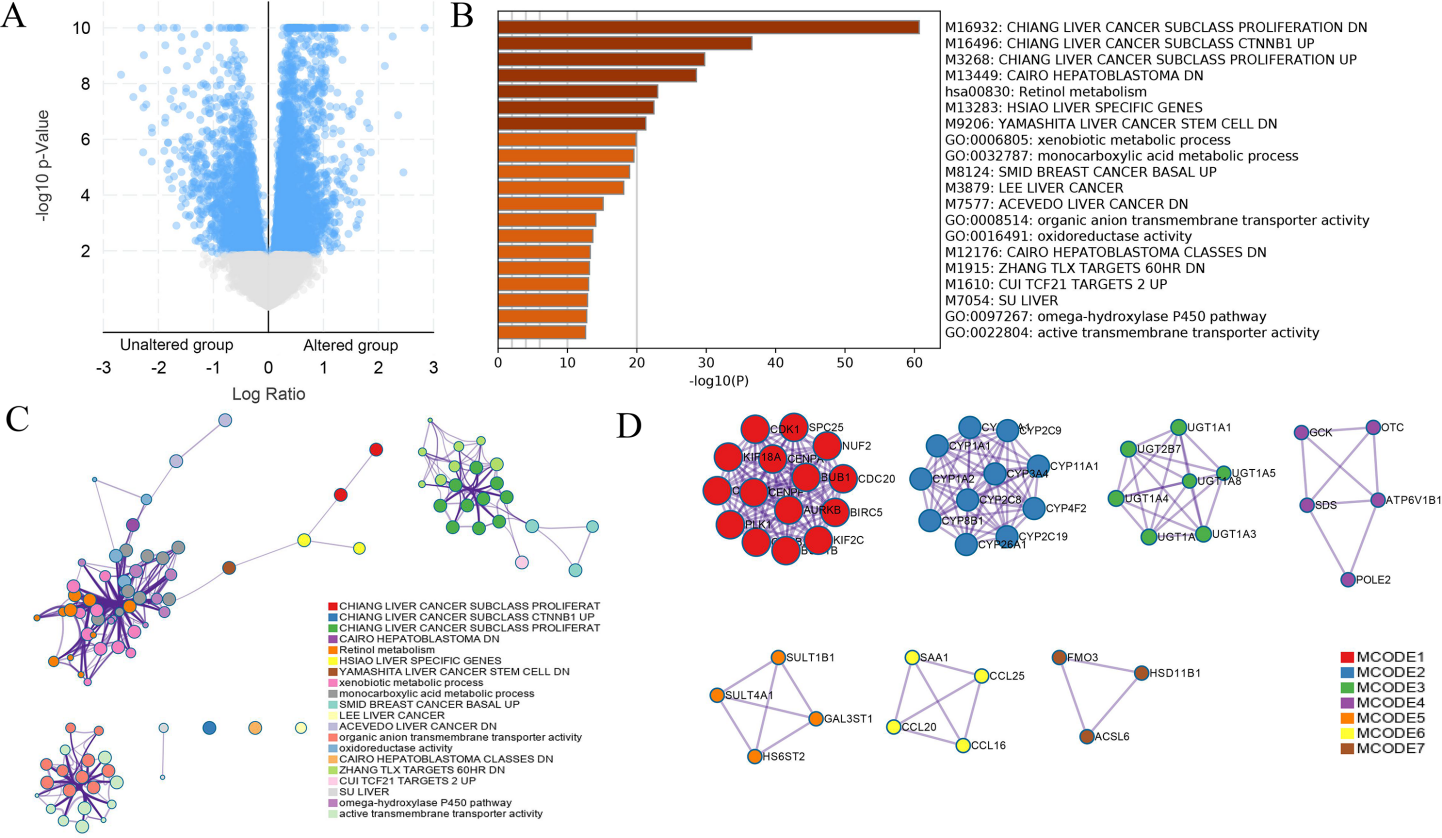
DEGs between HCC cases with altered and nonaltered *DUSP12*. (A) Volcano plot of DEGs between HCC cases with altered and nonaltered *DUSP12*. (B) Heatmap for selected terms (*P* < 0.05). (C) Network colored by cluster. (D) MCODEs of the PPI network. DEGs, differentially expressed genes; MCODEs, Minimal Common Oncology Data Elements; PPI, protein-protein network.

An online tool in the cBioPortal database was utilized to analyze the mutant status of *DUSP12* in the Liver Hepatocellular Carcinoma (TCGA, Firehose Legacy) dataset. The mutant frequency of *DUSP12* in HCC was 33.0% (Fig. 5A), which was composed mainly of amplification and high expression of mRNA (Fig. 5B). mRNA expression of *DUSP12* in HCC with amplification was higher than that in those without alteration (Fig. 5C). HCC patients with altered *DUSP12* had shorter OS than HCC patients with nonaltered *DUSP12* (Fig. 5D). CNV analyses revealed that mRNA expression of *DUSP12* was higher in HCC patients with *DUSP12*-amplification and *DUSP12*-gain patients than in those with *DUSP12*-shallow deletion and *DUSP12*-diploid (Fig. 6A). In general, mRNA expression of *DUSP12* was correlated negatively with methylation of the promoter region of *DUSP12* in 359 HCC samples from the TCGA-LIHC (Firehose Legacy) dataset (Fig. 6B). *DUSP12*-altered patients had a higher serum level of alpha fetoprotein (AFP) at procurement (Fig. 6C), fraction of genome altered (Fig. 6D) and worse histology grade in neoplasms (Fig. 6E). In addition, we screened 392 DEGs between *DUSP12*-altered patients and *DUSP12*-nonaltered patients with false discovery rate <0.05 and —log(ratio)—>1 (Fig. 7A). Analyses of functional enrichment and disease-related enrichment of these 392 DEGs revealed that these genes mainly took part in: “M61392: CHIANG LIVER CANCER SUBCLASS PROLIFERATION DN”; “M16496: CHIANG LIVER CANCER SUBCLASS CTNNB1 UP”; “M3268: CHIANG LIVER CANCER SUBCLASS PROLIFERATION UP” (Figs. 7B, 7C). Minimal Common Oncology Data Elements (MCODE) analyses revealed clustering of seven MCODEs (Fig. 7D). MCODE 1 mainly included proteins that took part in the cell cycle (e.g., cluster of differentiation (CD)K1, CDC20, PLK1), cell division or mitosis (e.g., AURKB, BIRC5, KIF2C, CENPA, CENPF). MCODE 2 mainly included cytochrome P450 (CYP) monooxygenase proteins that took part in various types of metabolism. MCODE 3 mainly included UDP-glucuronosyltransferase (UDPGT) proteins. MCODE 4 included GCK, OTC, SDS, POLE2 and ATP6V1B1. MCODE 5 included SULT4A1, SULT1B1, HS6ST2, and GAL3ST1. MCODE 6 mainly included C-C motif chemokine proteins. MCODE 7 included FMO3, ACSL6 and HSD11B1. MCODE 1 and MCODE 2 may play a critical part in the proliferation and biological activity of HCC cells. MCODE 6 may have a correlation with the infiltrations of immune cells in HCC samples.

### Analyses of *DUSP12* expression using the TIMER database

**Figure 8:**
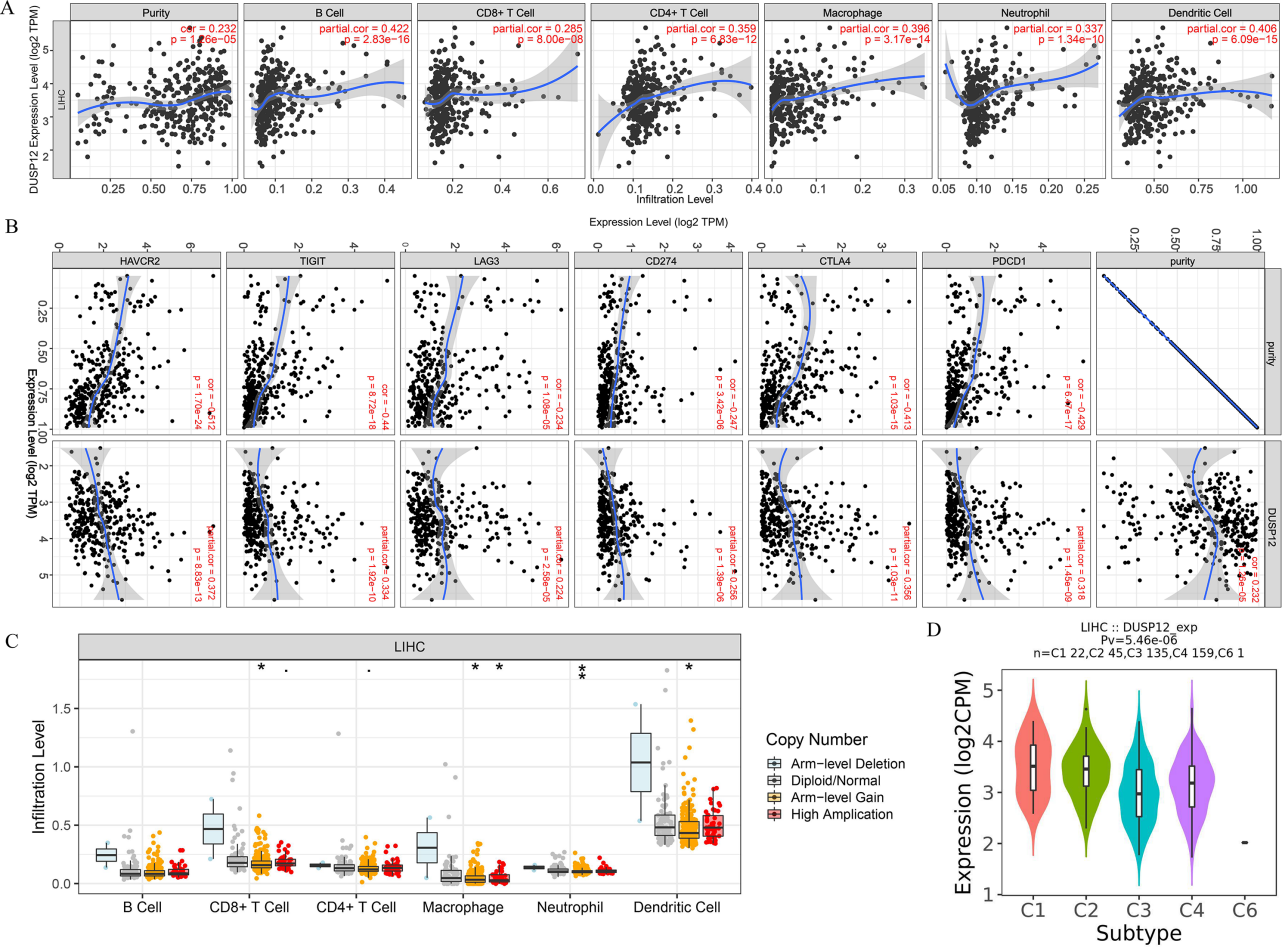
Correlation between *DUSP12* expression and number of tumor-infiltrating immune cells. (A) Pearson correlation between *DUSP12* expression with TIICs abundance. (B) Pearson correlation between *DUSP12* expression with immune-checkpoint moieties. (C) *DUSP12* expression in HCC cases with different copy numbers. (D) *DUSP12* expression in HCC cases with different immune subtypes. TIIC, tumor infiltrating immune cells. ^∗^*P* < 0.05, ^∗∗^*P* < 0.01.

**Figure 9:**
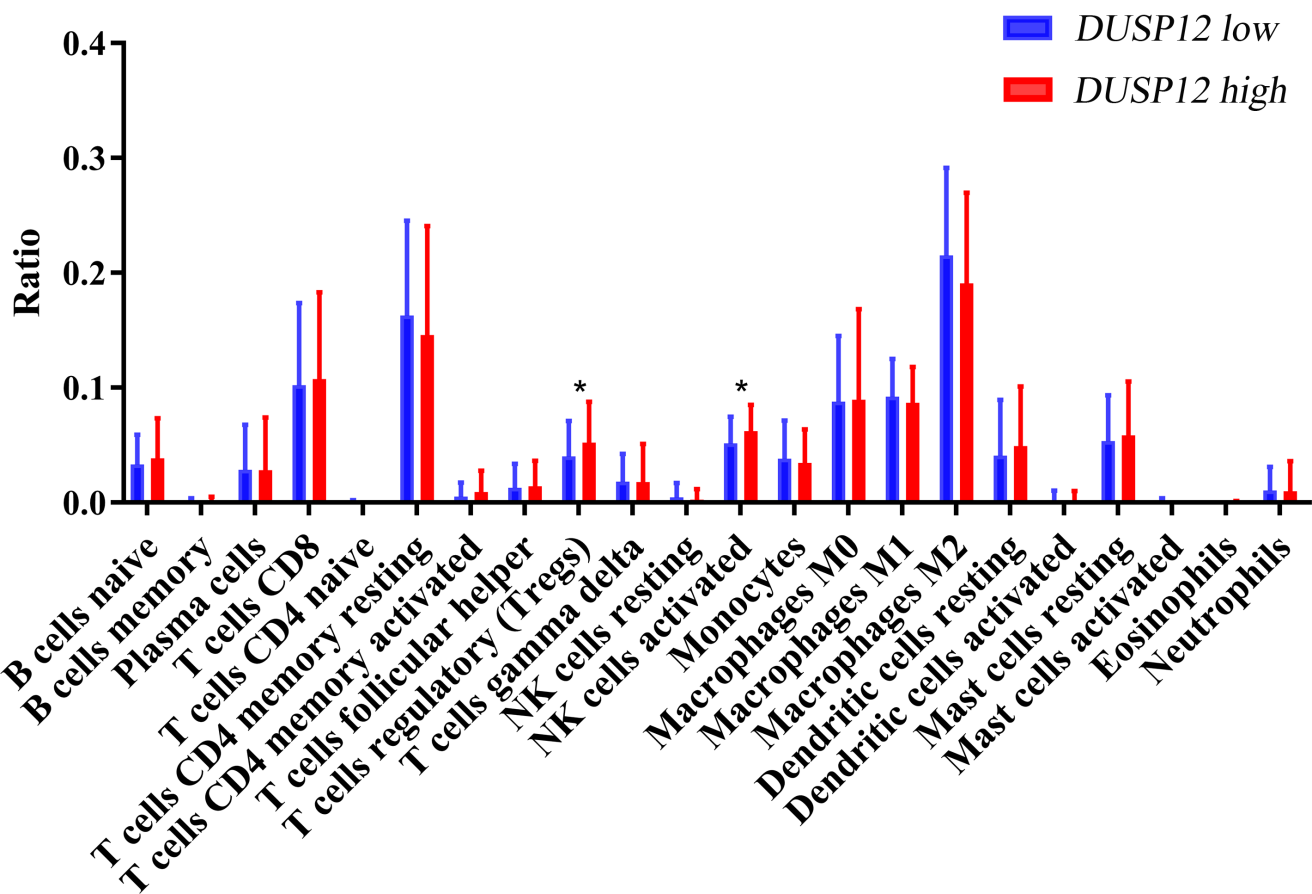
Ratio of various TIICs in HCC. ^∗^*P* < 0.05. TIICs, tumor infiltrating immune cells.

We explored the correlation of *DUSP12* expression with TIICs by TIMER database. *DUSP12* expression was moderately (partial correlation >0.2) positively correlated with the abundance of infiltrating B cells, CD4+ T cells, macrophages, neutrophils, and dendritic cells (Fig. 8A). Then, we explored the correlation between *DUSP12* and immune cells with other immune-infiltration analyzing methods (xCELL, EPIC, CIBERSORT). The result suggested that DUSP12 expression was moderately (partial correlation >0.2) positively correlated with the abundance of infiltrating B cells and CD4+ T cells regardless of the method employed (Table 1). *DUSP12* expression had a moderately positive correlation with the immune-checkpoint moieties HAVCR2, TIGIT, CTLA4 and PD-1 (Fig. 8B). The infiltration level of CD8+ T cells, macrophages, neutrophils, and dendritic cells was significantly different in *DUSP12* with different CNV (Fig. 8C). Furthermore, we explored mRNA expression of *DUSP12* in patients with different immune-subgroup liver cancer using the TISIDB. We revealed that the C1 (wound healing) subgroup had the highest *DUSP12* expression, whereas the C3 (inflammatory) and C6 (TGF-β dominant) subgroups had lower expression of *DUSP12* (Fig. 8D). Furthermore, groups with high expression of *DUSP12* had a higher ratio of T-regulatory cells (T_regs_) and activated natural-killer cells compared with those with low expression of *DUSP12* (Fig. 9).

**Table 1:** The correaltion of DUSP12 and immune cell with four differernt immune-infiltration analyzing methods (adj. *p* < 0.05).

Tools	infiltrates	rho	*p*	adj.p
xCELL	B cell memory_XCELL	0.144057569	0.0073615	0.043337621
	B cell_XCELL	0.199439884	0.000192613	0.002495049
	Class-switched memory B cell_XCELL	0.184646391	0.000567075	0.005704576
	Macrophage M2_XCELL	−0.514075954	1.15E−24	3.30E−21
	Macrophage_XCELL	−0.242486638	5.22E−06	0.000115083
	Myeloid dendritic cell activated_XCELL	0.197596364	0.00022132	0.002791875
	Plasmacytoid dendritic cell_XCELL	−0.148823775	0.005611022	0.036069056
	T cell CD4+ memory_XCELL	0.196441351	0.000241293	0.002953574
	T cell CD4+ Th2_XCELL	0.360960796	4.69E−12	5.17E−10
TIMER	B cell_TIMER	0.411173294	1.66E−15	3.66E−13
	Macrophage_TIMER	0.337986628	1.15E−10	8.78E−09
	Myeloid dendritic cell_TIMER	0.476711143	5.62E−21	4.03E−18
	Neutrophil_TIMER	0.246221689	3.69E−06	8.33E−05
	T cell CD4+_TIMER	0.298486339	1.57E−08	6.73E−07
EPIC	B cell_EPIC	0.162734334	0.002430024	0.019352438
	Macrophage_EPIC	−0.480988446	2.24E−21	2.14E−18
	T cell CD4+_EPIC	0.151948975	0.004676291	0.031694861
CIBERSORT	B cell memory_CIBERSORT-ABS	0.143292145	0.007684113	0.044686314
	B cell plasma_CIBERSORT-ABS	0.154562661	0.004005	0.027869748
	Macrophage M0_CIBERSORT	0.180630369	0.000749909	0.007003218
	Macrophage M0_CIBERSORT-ABS	0.305859665	6.63E−09	3.22E−07
	Macrophage M1_CIBERSORT-ABS	0.314940048	2.21E−09	1.19E−07
	Macrophage M2_CIBERSORT-ABS	0.349115494	2.52E−11	2.26E−09
	Myeloid dendritic cell resting_CIBERSORT	0.178434015	0.000871612	0.008087095
	Myeloid dendritic cell resting_CIBERSORT-ABS	0.21779062	4.51E−05	0.00073801
	T cell CD4+ memory resting_CIBERSORT-ABS	0.269145392	3.88E−07	1.10E−05
	T cell CD8+_CIBERSORT-ABS	0.186805703	0.000486791	0.005056632

### Knockdown of *DUSP12* expression decreases the proliferation and migration of Huh-7 cells

**Figure 10:**
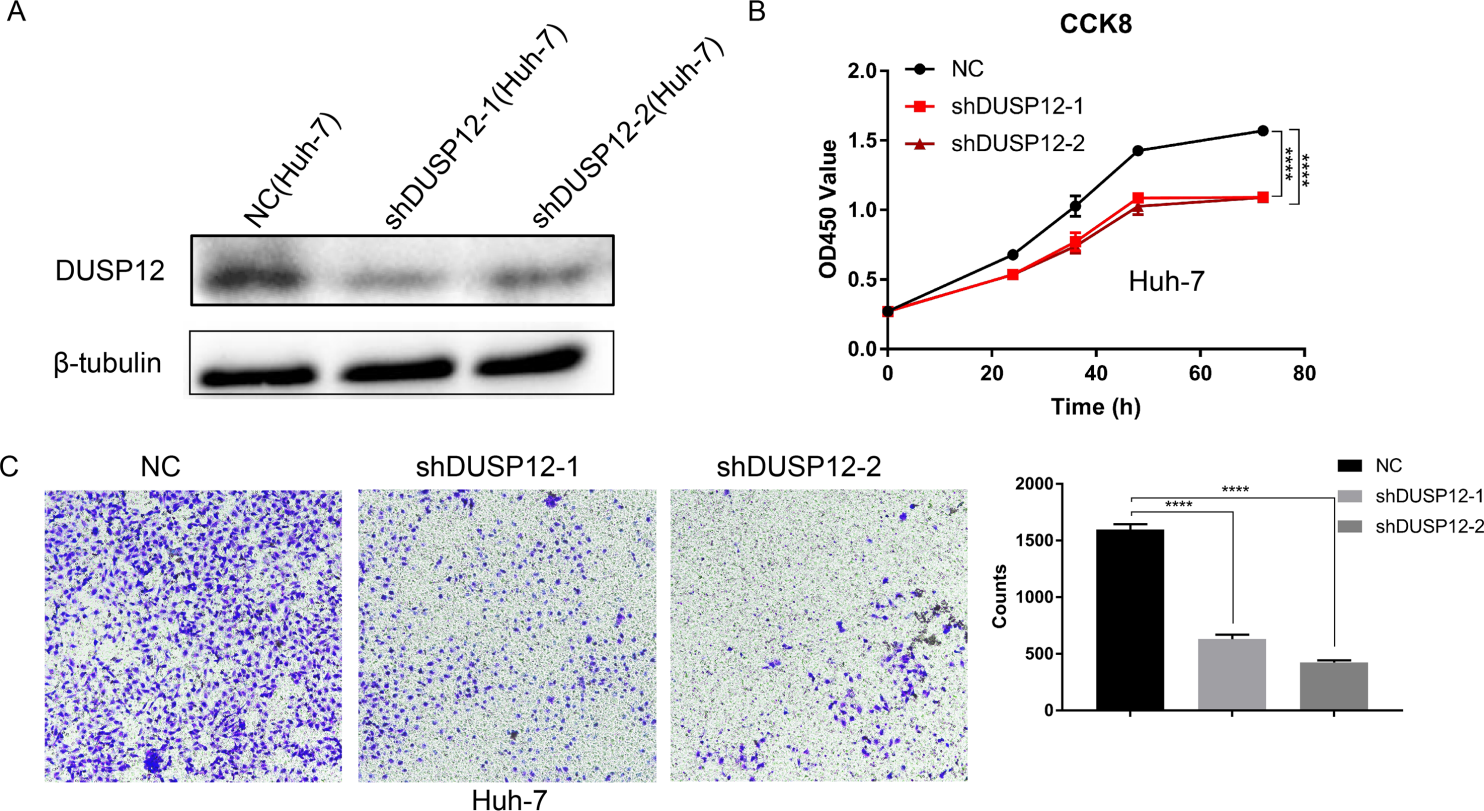
Knockdown of *DUSP12* expression reduces the proliferation and migration of Huh7 cells. (A) Knockdown of *DUSP12* expression in Huh7 cells. (B) Proliferation of cells according to the CCK8 assay. (C) Migration of cells according to the Transwell™ assay. ^∗∗∗∗^*P* < 0.0001.

We evaluated the probabilities of dependency of *DUSP12* in 22 HCC cell line types with data from CRISPR (DepMap 21Q2 Public + Score, CERES) cohort. The CERES scores of cell lines ranged from −0.04 to −0.47 while mean value was equal to −0.21 (Fig. S2). In general, a lower score meant that a gene is more likely to be essential in a given cell line and a score <0 meant that down-regulation of a gene may inhibit the proliferation of a given cell line. Human liver-cancer cells (Huh-7) were transfected with a specific shRNA for *DUSP12* (sh*DUSP12*) and a nonspecific shRNA (NC) (Fig. 10A). CCK8 and Transwell assays were utilized to evaluate the ability of cells to proliferate and migrate. Knockdown of *DUSP12* expression led to the reduced proliferation and motility of Huh7 cells (Figs. 10B, 10C).

## Discussion

We propose that expression of *DUSP12*, a member of the PTP family, was different in HCC tissues and normal liver tissues in multiple datasets. In addition, the clinical features of HCC patients had a strong relationship with *DUSP12* expression, including ethnicity, TNM stage, histology grade, and P53-mutant status. Survival analyses using the Kaplan–Meier method demonstrated that HCC patients with higher expression of *DUSP12* had shorter survival than those with lower expression of *DUSP12*.

A PPI network containing *DUSP12* and 20 genes in liver-cancer samples in HCCDB was constructed. These genes, including ubiquitin-conjugating enzyme E2T (UBE2T), ILF2, SETDB1, CCT3, and UFC1, were engaged mainly in histone methylation, cullin RING ubiquitin ligase complexes, nuclear specks, and ubiquitin-like protein transferase activity. UBE2T has been demonstrated to promote the growth of HCC cells by regulating ubiquitination of P53, transition of the G2/M phase of the cell cycle, and the protein kinase B signaling pathway (Liu et al., 2019a; Liu et al., 2017; Wei et al., 2019). IL-F2 is a transcription factor which regulates the growth of HCC cells by controlling mRNA expression of apoptosis-related proteins (Cheng et al., 2016). Setdb1 is a histone methyltransferase that also regulates the growth of HCC cells by P53 methylation (Fei et al., 2015). CCT3 shows high expression in liver cancer, and leads to short survival (Liu et al., 2019b). CCT3 triggers expression of YAP and TFCP2 to regulate HCC tumorigenesis (Liu et al., 2019b). Hence, *DUSP12* may have a critical role in the processes mentioned above by interacting with proteins in this network.

The mutant status of *DUSP12* in HCC patients was determined by utilizing an online tool in the cBioPortal database. We discovered that nearly one-third of HCC patients suffered a *DUSP12* mutation. Most of them experienced amplification and higher mRNA expression of *DUSP12*. HCC patients with a *DUSP12* mutation had shorter survival, higher serum level of AFP, and worse histology grade than those of patients with wild-type *DUSP12*. Taken together, these findings suggest the probability of an intimate correlation between *DUSP12* mutation and the pathology and prognosis of *DUSP12* in HCC.

We also identified 392 DEGs between HCC patients with altered *DUSP12* and HCC patients with nonaltered *DUSP12*. These DEGs mainly regulated the tumorigenesis and proliferation of HCC cells. MCODE analyses revealed clustering of seven MCODEs. Among them, MCODE 1 mainly comprised the mutations of CDK1, KIF2C, KIF18A, CENPA, and PLK1, which take part in the tumorigenesis and progression of liver cancer (Jung et al., 2019; Komatsu et al., 2009; Li et al., 2020; Li et al., 2011; Long et al., 2018; Luo et al., 2018; Wu et al., 2018; Zhang et al., 2015). MCODE 2 was composed mainly of members belonging to the CYP enzyme superfamily, which has a critical role in drug metabolism or chemical metabolism in the liver (Agundez, 2004). Agundez and colleagues found that the activity of CYP enzymes was closely associated with the risk of liver cancer (Agundez, 2004). MCODE 3 mainly included UDPGT proteins that take part in conjugation and subsequent elimination of potentially toxic xenobiotics and endogenous compounds. With regard to the components of MCODE 4, GCK helps to facilitate the uptake and conversion of glucose by acting as an insulin-sensitive determinant of hepatic-glucose usage (Velho et al., 1996) and OTC catalyzes the second step of the urea cycle (Horwich et al., 1984). With respect to the components of MCODE 6, it has been reported that CCL20 facilitates T_reg_ activity in advanced HCC (Li & Liu, 2016).

We explored the association between TICCs and *DUSP12* expression. *DUSP12* expression was positively correlated with the abundance of tumor-infiltrating CD4+ T cells, macrophages, neutrophils, dendritic cells, and expression of the immune-checkpoint moieties HARVC2, TIGIT, CTLA4 and PDCD1. Increased expression of these immune-checkpoint moieties denoted a phenotype of liver cancer associated with a poor outcome. Use of TISIDB revealed that patients in the C3 immune-subgroup had the longest survival (Thorsson et al., 2018) and had the lowest expression of *DUSP12* (except the C6 group, which contained only one patient). Furthermore, we investigated the ratio of various types of immune cells in total TIICs between a *DUSP12*-high-expression group and *DUSP12*-low-expression group. We showed that the infiltrating abundance of T_regs_ was higher in *DUSP12*-high-expression HCC samples compared with that in *DUSP12*-low-expression HCC samples. T_regs_ can inhibit the anti-tumor effects of immune cells and facilitate immune evasion by liver-cancer cells (Jiang et al., 2017; Langhans et al., 2019). This phenomenon may be one of the reasons why patients with high expression of *DUSP12* experience rapid progression of disease and have a shorter survival time.

## Conclusions

We propose that *DUSP12* has a critical role in the tumorigenesis and progression of HCC. *DUSP12* could be a potential target for curing liver cancer.

##  Supplemental Information

10.7717/peerj.11929/supp-1 Supplemental Information 1 DUSP12 expression in HCC and normal liver tissues in ICGC-LIRI-JP and GSE14520 cohorts DUSP12 expression in HCC and normal liver tissues in ICGC-LIRI-JP and GSE14520 cohorts. (A) ICGC-LIRI-JP. (B) GSE14520. ****P < 0.0001.

10.7717/peerj.11929/supp-2 Supplemental Information 2 The top positively correlated genes in TCGA dataset for DUSP12 (Pearson correlation coefficient > = 0.3) The top positively correlated genes in TCGA dataset for DUSP12 (Pearson correlation coefficient > = 0.3).

10.7717/peerj.11929/supp-3 Supplemental Information 3 Dependency of DUSP12 in HCC cell lines Dependency of DUSP12 in HCC cell lines

10.7717/peerj.11929/supp-4 Supplemental Information 4 The top negatively correlated genes in TCGA dataset for DUSP12 (Pearson correlation coefficient < = −0.3) The top negatively correlated genes in TCGA dataset for DUSP12 (Pearson correlation coefficient < = −0.3).

10.7717/peerj.11929/supp-5 Supplemental Information 5 Raw data of CCK8 assay

10.7717/peerj.11929/supp-6 Supplemental Information 6 Raw data (WB-internal control)

10.7717/peerj.11929/supp-7 Supplemental Information 7 Raw data (WB-DUSP12)

10.7717/peerj.11929/supp-8 Supplemental Information 8 Cell couts of transwell assay (imageJ)

## Additional Information and Declarations

### Data Availability

The following information was supplied regarding data availability: The CCK8 data, Transwell data, and western blots are available in the Supplemental Files.
