# Supplementary figures and images for: DUSP12 regulates the tumorigenesis and prognosis of hepatocellular carcinoma
Source: PeerJ. 2021 Aug 3;9:e11929. doi: 10.7717/peerj.11929 (PMC8344690; doi:10.7717/peerj.11929)

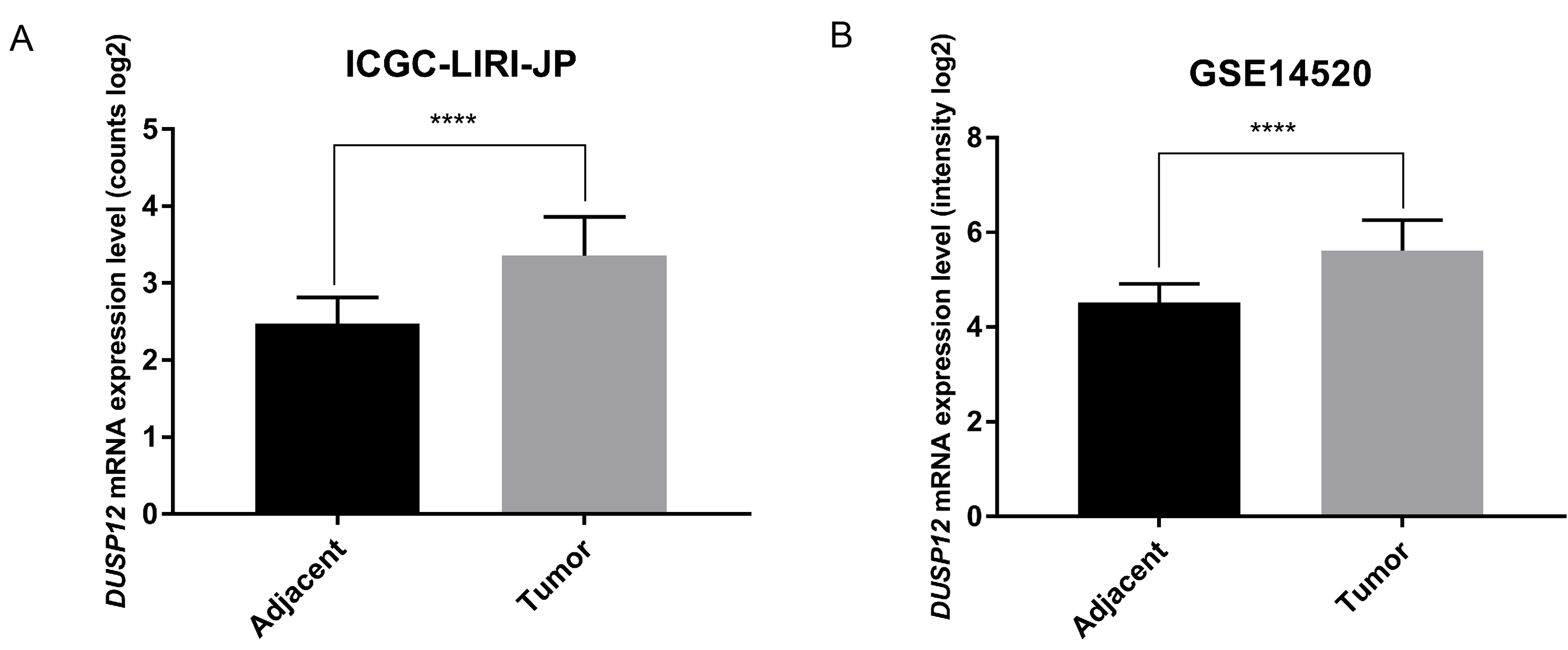

Supplement: Supplemental Information 1 — DUSP12 expression in HCC and normal liver tissues in ICGC-LIRI-JP and GSE14520 cohorts. (A) ICGC-LIRI-JP. (B) GSE14520. ****P < 0.0001. [file peerj-09-11929-s001.png]

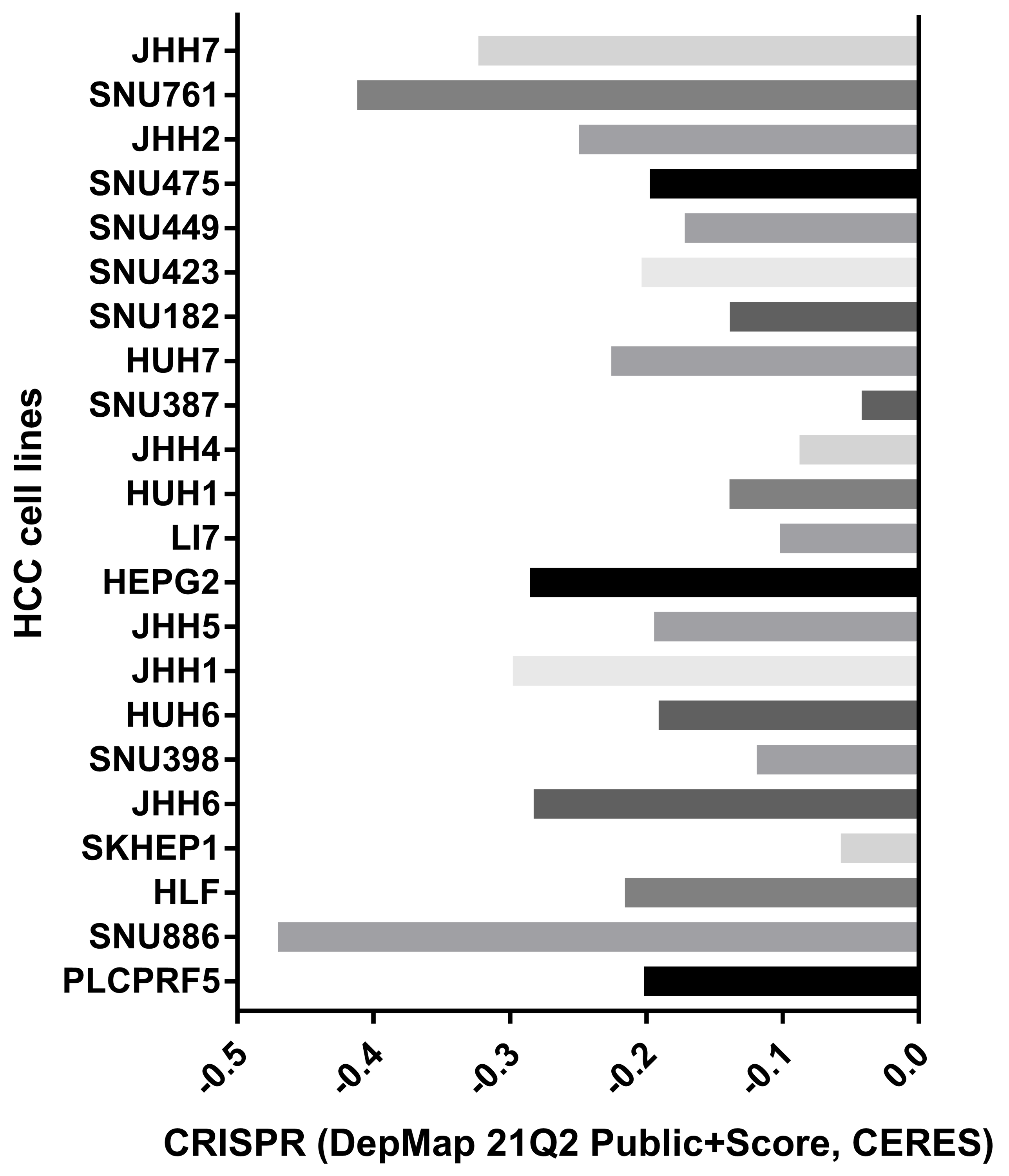

Supplement: Supplemental Information 3 — Dependency of DUSP12 in HCC cell lines [file peerj-09-11929-s003.png]

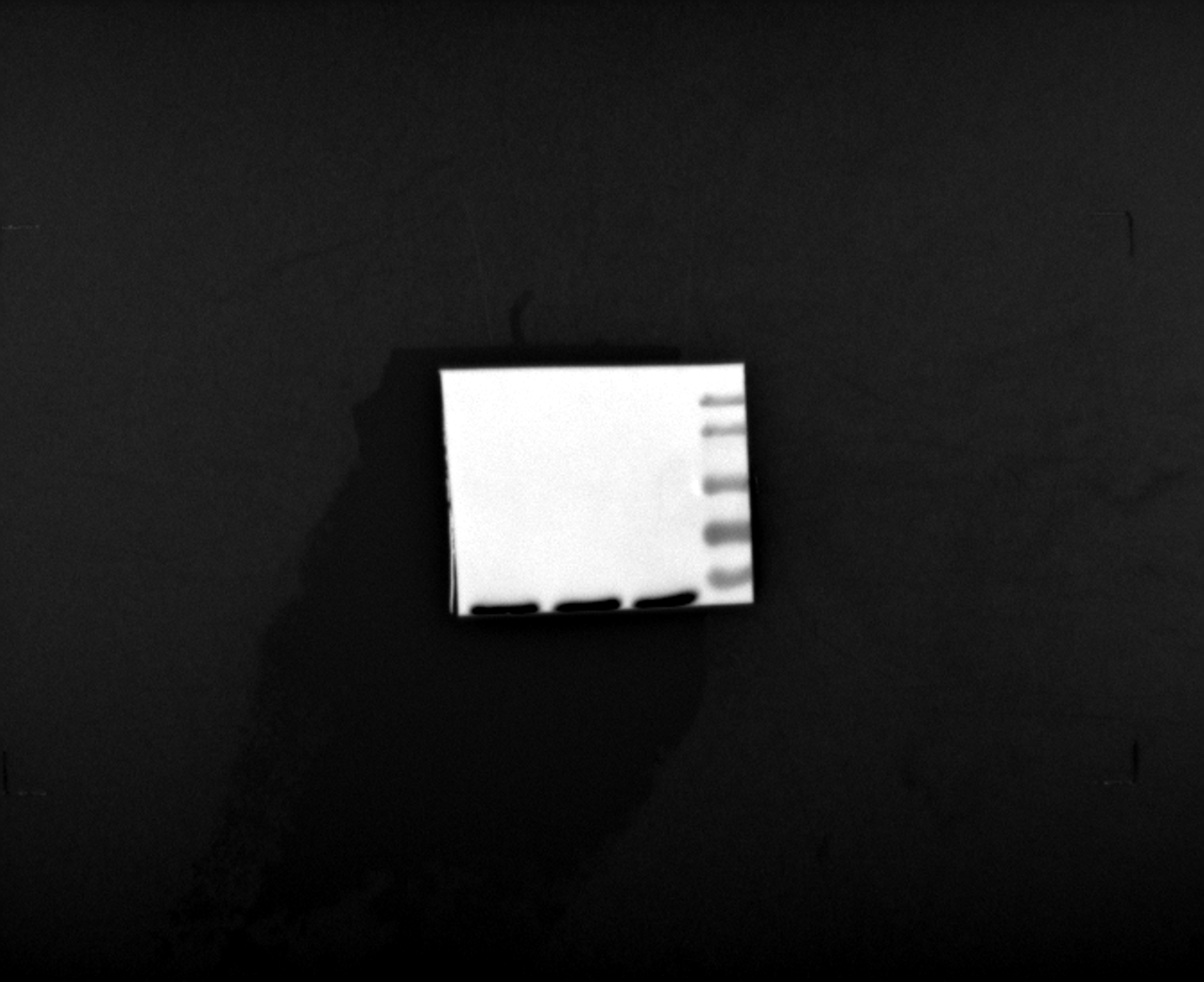

Supplement: Supplemental Information 6 [file peerj-09-11929-s006.png]

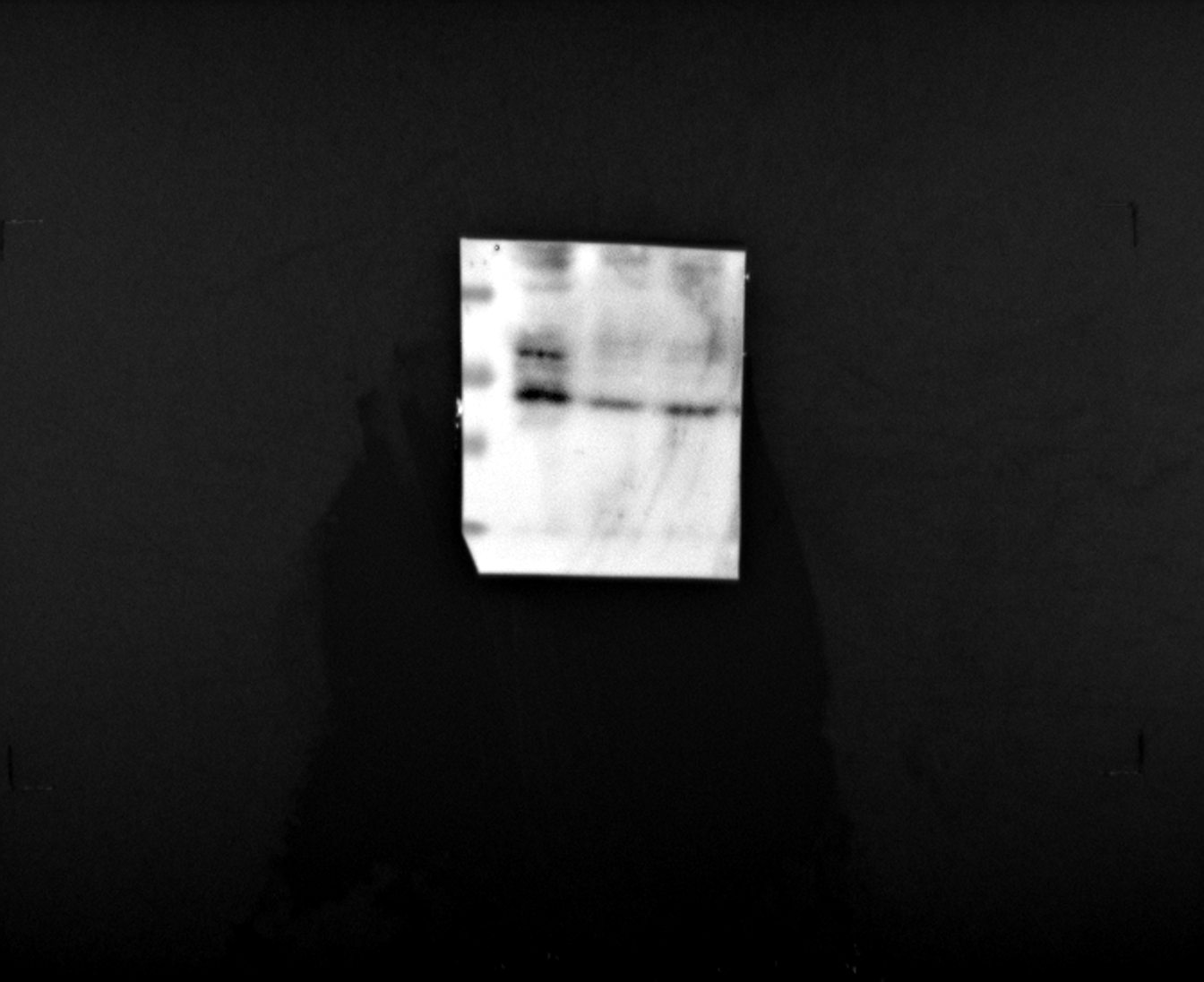

Supplement: Supplemental Information 7 [file peerj-09-11929-s007.png]
